# Atypical Neural Responses to Native and Non-Native Language in Infants at High Likelihood for Developing Autism

**DOI:** 10.21203/rs.3.rs-5190659/v1

**Published:** 2024-12-03

**Authors:** Lauren Wagner, Megan Banchik, Tawny Tsang, Nana J. Okada, Rebecca Altshuler, Nicole McDonald, Susan Y. Bookheimer, Shafali S. Jeste, Shulamite A. Green, Mirella Dapretto

**Affiliations:** University of California, Los Angeles; University of California, Los Angeles; Ahmanson-Lovelace Brain Mapping Center; Harvard University; University of Colorado Anschutz Medical Campus; University of California, Los Angeles; University of California, Los Angeles; Children’s Hospital of Los Angeles; University of California, Los Angeles; University of California, Los Angeles

**Keywords:** Infant, autism, fMRI, language, native language

## Abstract

**Background:**

Language difficulties are common in autism spectrum disorder (ASD), a neurodevelopmental condition characterized by impairments in social communication as well as restricted and repetitive behaviors. Amongst infant siblings of children with an ASD diagnosis – who are at higher likelihood for developing ASD – a high proportion also show difficulties and delays in language acquisition.

**Methods:**

In this study, we used functional magnetic resonance imaging (fMRI) to examine atypicalities associated with language processing in 9-month-old infants at high (HL) and typical (TL) familial likelihood for ASD. Infants were presented with native (English) and novel (Japanese) speech while sleeping naturally in the scanner. Whole-brain and *a priori* region-of-interest analyses were conducted to evaluate neural differences in language processing based on likelihood group and language condition.

**Results:**

HL infants showed attenuated responses to speech in general, particularly in left temporal language areas, as well as a lack of neural discrimination between the native and novel languages compared to the TL group. Importantly, we also demonstrate that HL infants show distinctly atypical patterns of greater rightward lateralization for speech processing. Limitations: The sample size, particularly for the TL group, is relatively modest due to the challenges inherent to collecting auditory stimulus-evoked data from sleeping participants, as well as retention and follow-up difficulties posed by the COVID-19 pandemic. Additionally, the groups were not matched on some demographic variables; however, the present findings held even after accounting for these differences.

**Conclusions:**

To our knowledge, this is the first fMRI study to directly measure autism-associated atypicalities in native language uptake during infancy. These findings provide a better understanding of the neurodevelopmental underpinnings of language delay in ASD, which is a prerequisite step for developing earlier and more effective interventions for autistic children and HL siblings who experience language impairments.

## Background

Language difficulties, including speech delays and impairments in receptive language, are the most commonly reported first signs of autism spectrum disorder (1, 2) (ASD), a neurodevelopmental condition that affects approximately one in 36 children in the United States (3). Indeed, communication difficulties are part of the core criteria for autism and approximately 25% of autistic individuals are minimally verbal or entirely nonverbal (4). These prevalent, and often profound, impairments underscore the need to understand how early brain development contributes to atypical language outcomes in autism. Due to the vast heterogeneity that characterizes the onset, developmental course, and severity of core ASD symptoms (5), studies searching for early-life biomarkers of autism have continued to investigate how early atypical development cascades into complex symptom profiles.

Human infants begin to acquire language before birth, a process that involves many months of uptake of the prosodic contours – such as rhythm, stress, and pitch – of the language spoken *ex utero* (6, 7). Soon after birth, this uptake is displayed when neonates show a preference for their native language over a novel language (8). In mid- to late infancy, this familiarity preference is then supplanted by a preference for novel stimuli (9), which roughly coincides with the process of phonemic “perceptual narrowing,” in which infants lose the ability to discriminate between nonnative sounds between six and 10 months (10, 11). As an inherently social process, language acquisition depends heavily on joint attention and affect sharing skills, both of which are perturbed in ASD (12, 13). Indeed, language impairments associated with autism, such as a failure for infants to orient to their own name (14), can be detected well before a clinical ASD diagnosis is obtained. A recent review of pre-speech milestones in infants later diagnosed with ASD (15) summarized the behavioral atypicalities that can be found within the first year of life. These included lower rates of vocalizing (16), vocalizations with simpler intonations and acoustic contours (17), and, in some cases, a failure to coo at developmentally appropriate ages (18). By approximately 12 months of age, infants later diagnosed with ASD also exhibit lower rates of canonical babbling (19, 20), an important speech milestone.

Despite these promising behavioral markers, infants who later receive an ASD diagnosis cannot yet be reliably identified within the first year of life with current linguistic assessment tools. Although ASD can be diagnosed as early as 18 months of age (21), the median age of diagnosis in the United States lags behind, at an average of three to five years (22, 23). Closing this diagnostic gap is critical for children to receive earlier access to interventions, which is known to improve linguistic and cognitive outcomes (24). Moreover, understanding the considerable variability in the severity and developmental course of language impairments in ASD will help inform more individualized interventions for affected children.

Consistent with the behavioral literature in infancy, pre-partum studies have demonstrated that typically developing fetuses show increased heartrate, a physiological response associated with preferential attention, when presented with a novel language (6). Likewise, fetuses also show increased heartrate (6) and activation in temporal language areas when hearing their mother’s voice compared to a stranger’s voice (7). Further, activity in frontal and temporal regions – associated with language processing in adults – has been demonstrated in two-day-old neonates passively exposed to speech (25). Collectively, these studies not only show that native language acquisition begins in the earliest stages of human development, but that the early antecedents of the adult language network (26) are already in place.

Atypicalities in the brain’s responses to speech have been broadly documented in toddlers and young children with autism. Canonical activity in temporal language areas is notably diminished in autistic toddlers (27), particularly those with delayed language (28). Moreover, responses to speech, which are left-lateralized in most individuals, are atypically right-lateralized in toddlers with ASD (29). Some of these differences may stem from early deficits in attention to socially relevant and/or affective speech, such as infant-directed speech. Indeed, recent work shows that autistic toddlers with poor language skills show weaker brain responses in temporal cortices when hearing infant-directed speech (27), providing a neural correlate for the diminished attention to early affective speech – and language in general – seen in ASD. Additionally, toddlers with autism show reduced left-hemisphere specialization for language processing compared to controls (29, 30), with atypical rightward lateralization of language-related white matter tracts detected in infants as young as six weeks (31). Newer evidence also indicates that the cerebellum (known to be involved in language processing (32)) may play a role in language delays associated with ASD. Altered connectivity between the right crus I of the cerebellum – often implicated in language function (33) – and the cortex has been observed in language-delayed infants who also later developed ASD and/or other developmental concerns (34).

A growing body of work has focused on infancy, before autism can be reliably diagnosed, by prospectively studying infants at high likelihood (HL) for ASD based on family history. HL infants, who have an older sibling with a confirmed diagnosis, have a one in five chance of receiving an ASD diagnosis by age three (35). HL infants have shown reduced neural specialization for language (36), among other atypical brain responses (37), during passive exposure to speech. In another paradigm that examined implicit language learning, HL infants, particularly those with greater ASD symptoms, showed reductions in neural activity associated with statistical language learning at nine months of age (38). These studies show that passive activation paradigms can consistently uncover the neural traces of language atypicalities in infants, including differences associated with autism likelihood and later symptoms. However, prior studies of speech processing in infants have either compared brain responses to native language versus non-speech sounds or backwards speech. To date, no studies have directly compared neural responses to native versus non-native language in HL infants even though doing so would provide an index of native language uptake at a very early age.

In this study, we used functional magnetic resonance imaging (fMRI) to index early atypicalities in native language acquisition that may be related to ASD. Specifically, we compared infant brain responses to native versus non-native speech at nine months of age, when infants are expected to show a novelty preference for a new language, in infants at varying likelihood for ASD: HL infants and “typical likelihood” (TL) controls. Based on prior work (29, 36), we expected that the HL group would be characterized by diminished responses to speech stimuli in language networks, including the cerebellum, with reduced neural differentiation between native and non-native languages. Furthermore, we expected the HL group to show atypically lateralized processing of native speech, characterized by more bilateral language processing, or even a rightward bias, given evidence that right-lateralized language processing may be compensatory in older autistic individuals (39).

## Materials & Methods

### Participants

Participants were enrolled as part of a longitudinal study of behavioral and neural markers of ASD as part of UCLA’s Autism Center for Excellence. Based on family history of ASD, infants were assigned to the following groups: “High Likelihood” (HL) infants had at least one older sibling with a confirmed clinical diagnosis of autism, and “Typical Likelihood” (TL) infants had no first or second-degree family members with ASD or another known neurodevelopmental disorder. For both groups, exclusionary criteria included: 1) genetic or neurological conditions associated with ASD (e.g., fragile X syndrome, tuberous sclerosis), 2) chronic developmental condition or perinatal insult, 3) severe visual, hearing, or motor impairment, and 4) contraindication for MRI. Informed consent was obtained from participants’ parents or legal guardians, and all study protocols were approved by UCLA’s Institutional Review Board.

For this particular study, participants were required to have at-home English exposure of at least 50%, with 0% exposure to Japanese. Although some infants may be acquiring multiple “first” languages, this ensured that English could be considered the native language of all participants, with Japanese remaining entirely novel. In cases where sibling pairs were recruited, we retained only one sibling (the sibling with lowest head motion during fMRI and/or with the most behavioral data available) in order to preserve independence of observations. These criteria yielded a sample of 63 infants (38 HL, 25 TL). Out of these, we excluded two TL participants due to scanner artifacts, two HL participants due to motion artifacts, and 10 participants (six HL, four TL) due to lack of any response in primary auditory cortices even at extremely liberal thresholds (Z > 1.1, uncorrected) to guard against the possibility of a stimulus presentation failure. Therefore, the final imaging sample consisted of 49 infants (30 HL, 19 TL). The HL and TL groups did not significantly differ on age at scan, race, ethnicity, exposure to English, or head motion during the MRI scan, but the groups significantly differed on sex, maternal education, and birth order (Table 1). As these variables can affect language development, post-hoc linear regressions were conducted to ensure that group differences held when controlling for these potential confounds (see Supplementary Information).

### Behavioral Measures

Several behavioral assessments were administered at 12 and 36 months of age to evaluate the development of social and language skills, as well as ASD symptomatology. The Mullen Scales of Early Learning (40) (MSEL) were administered at 12 and 36 months of age to index five components of early development: receptive and expressive language, fine and gross motor function, and visual reception. Together, these subscales (excluding gross motor) are combined into an Early Learning Composite (ELC) score. The Vineland Adaptive Behavior Scales, Second Edition (41) (VABS-II), a parental interview measure that assesses four domains including communication, daily living, socialization, and motor skills, was administered at 12 and 36 months. Here, we focused on the communication and socialization subscales. Early lexical development was indexed using the MacArthur-Bates Communicative Development Inventory (42) (MCDI), tracking the number of words understood and comprehended at 12 months, and the number of words produced at 36 months. The Early Social Communication Scales (43) (ESCS), a play-based, structured assessment of nonverbal social communication, was administered at 12 months to measure the rate per minute of responding to joint attention (RJA). Early signs of autism symptomatology were assessed using the Autism Observation Scale for Infants (44) (AOSI) at 12 months, with the Autism Diagnostic Observation Schedule-2nd Edition (45) (ADOS-2) collected at 36 months. At the 36-month behavioral visit, 26 HL and 16 TL participants underwent a clinical assessment to determine outcome classification: Typically Developing, Autism Spectrum Disorder, or Other Concerns (speech/language delay, subclinical ASD symptoms, or other developmental delays as assessed by a licensed UCLA psychologist).

### fMRI Paradigm

Following a traditional block design, speech stimuli were presented in alternating segments of native (English) and novel (Japanese) speech interspersed with 12 seconds of silence, during natural sleep. Each language was presented eight times, for a total of 2.4 minutes of exposure to each language condition. These stimuli, previously used in behavioral studies of native language preference in infants (46), were recorded by different female native speakers of each language and were matched on important acoustic features, including duration, pitch, pitch range, intensity, and peak amplitude to ensure that discrimination was based solely upon the prosodic, rhythmic, and phonological differences between English and Japanese. The recordings were delivered via Optoacoustics MRI-compatible headphones, which also served to dampen ambient scanner noise. The order of stimulus presentation (English versus Japanese blocks) was balanced between likelihood groups.

### MRI Data Acquisition

MRI scans were collected while infant participants were sleeping naturally, either on a Siemens Trio scanner (12-channel head coil) or, after an upgrade to the scanning facilities, a Siemens Prisma scanner (32-channel head coil). Scanner was therefore included as a nuisance covariate in group-level analyses. The data collection approach was based on recommended guidelines for neuroimaging in young infants (47, 48). A scout localizing scan was used for slice prescription. Matched bandwidth T2-weighted high-resolution echo planar images were acquired co-planar to the functional scans to ensure identical distortion characteristics to the fMRI scans: TR = 5000ms, TE = 34ms, matrix size = 128×128, FOV = 192mm, 34 slices, 1.5mm in-plane resolution, with 4mm-thick axial slices. The native language preference paradigm was acquired with a T2*-weighted functional sequence: TR = 3000ms, TE = 28ms, matrix size = 64×64, FOV = 192mm, 34 slices, 3mm in-plane resolution, with 4mm-thick axial slices.

MRI scans were conducted in the evening. To help participants sleep, parents were encouraged to follow the infant’s bedtime routine during the study visit and to swaddle or rock their child to sleep. Once asleep, infants were then transferred to the scanner bed, which was padded with linens and cushions. Silicone earplugs specially designed for infants and MiniMuffs Neonatal Noise Attenuators (Natus Medical, Inc., San Carlos, CA) were used for hearing protection underneath the stimulus presentation headphones. Noise suppression was not used. Infants were secured to the bed with a Velcro strap underneath a weighted blanket, and a member of the study staff remained in the scanner room with the infant at all times to monitor for signs of movement, wakefulness, or distress.

### fMRI Preprocessing

MRI scans were preprocessed using FMRIB’s Software Library version 5.0.11 (49). Functional scans underwent rigid-body motion correction, while structural scans underwent skull stripping with manual correction using FSL’s Brain Extraction Tool. Functional scans were co-registered to the infant’s own high-resolution T2-weighted anatomical scan before being registered to a 1-year standard template (50) as in previous studies from our lab (38, 51, 52). Registration at both steps was performed with a 12-parameter affine transformation, and registration was manually inspected for quality assurance. Data were spatially smoothed using a 6-mm Gaussian kernel and underwent 4D mean intensity normalization.

### fMRI Analysis

FSL FEAT was used to model BOLD responses to each condition of interest with respect to baseline, as well as to each other (English > Japanese, and Japanese > English), at the single-subject level. Motion-contaminated volumes, identified as outliers on FSL’s *dvars* metric (ΔRMS intensity > 50), were statistically censored from the analyses at the single-subject level(53). Group-level analyses were conducted with a mixed effects linear model in FSL FEAT (FLAME 1 + 2) to generate group activation maps in common space, and to conduct contrasts between likelihood groups, languages, and their interaction. Although the proportion of participants who underwent MRI on each scanner did not differ between the two groups (χ^2^ = 0.224, p = 0.636; Trio: 16 HL, 11 TL; Prisma: 14 HL, eight TL) a nuisance covariate for the effect of scanner was also included in the group-level model. Within- and between-group contrasts in response to English and Japanese were statistically thresholded at Z > 2.3, cluster-corrected for multiple comparisons (P < 0.05, based on Gaussian random field theory); however, we deemed significant and report only cluster peaks that surpass a threshold of Z > 3.1 (Table 2). Between-group and between-language contrasts were masked by clusters that displayed significant activation to either language condition, in either group based on the within-group activation maps.

### ROI and Laterality Analyses

To hone in on group differences in canonical language regions and to examine language lateralization, we complemented the whole-brain analytical approach by employing a more targeted region-of-interest (ROI) analysis. We selected *a priori* regions of interest (ROIs) canonically associated with language processing (26, 54), including the superior and middle temporal gyri, as well as the inferior frontal gyrus. In light of the growing evidence of the cerebellum’s role in language (32), we also considered two regions of the cerebellum known to be involved in language processing (54, 55): crus I and lobule VI. Activity in these ROIs was examined using two different metrics. First, to quantify activation strength for each ROI, for each infant we extracted parameter estimates from all voxels showing significant activation in either group and in either language. Second, the spatial extent of significant activation, quantified by the number of voxels with significant activation, was examined within our selected ROIs. This metric was calculated as the proportion of significant voxels within each ROI, extracted from each participant’s single-subject level data (thresholded at Z > 1.7 to account for individual differences in strength of activation). Mixed analyses of covariance (ANCOVAs) were then used to model these two metrics (activation strength and cluster size) with likelihood group as a between-group predictor, ROI, language condition and hemisphere as within-group predictors, and scanner as a covariate of no interest.

Furthermore, given that language processing has been found to be atypically lateralized in toddlers with ASD (29, 30), these two metrics were used to interrogate the role of laterality more directly. A laterality index was calculated for each ROI using a standard laterality formula defined as the difference in values in the left and right hemispheres, divided by the sum of values in both left and right hemispheres: *(L – R)/(L+R)*. The laterality indices of activation strength and cluster size were then examined as outcome variables in mixed ANCOVAs, which contained likelihood group as a between-group predictor, language condition and ROI as within-group predictors, and scanner as a covariate of no interest.

## Results

### Behavioral Results by Likelihood Group

For the infants who provided neuroimaging data, at 12 months of age the TL group had significantly higher scores, compared to the HL group, on the MSEL receptive language (p = 0.004) and fine motor (p = 0.004) subscales, as well as higher rates of responding to joint attention (p < 0.001) and the VABS-II communication subscale (p = 0.02). At 12 months, there were no group differences for the expressive language, gross motor, and visual reception MSEL subscales, nor on the number of words understood or produced as measured by the MCDI. By 36 months, the TL group scored significantly higher than the HL group on both the MSEL receptive and expressive language subscales (p = 0.0003 and p = 0.002, respectively), as well as the visual reception subscale (p = 0.02), but not the fine motor subscale. The TL group also had higher scores on the VABS-II communication (p < 0.001) and socialization subscales (p < 0.001). HL infants trended higher in ASD symptomatology (p = 0.08) as measured by the AOSI at 12 months, but there were no significant group differences in ASD symptomatology as measured by the ADOS-2 at 36 months. By 36 months, six HL infants received an ASD diagnosis, compared to none in the TL group (χ^2^ = 3.78, p = 0.05). We also found significantly higher rates of both ASD diagnosis and Other Concerns (language delay, intellectual delay, etc.) among the HL group (χ^2^ = 8.76, p = 0.013). Full demographic and behavioral data are reported in Table 1.

### Neuroimaging Group Results

**Figure 1:**
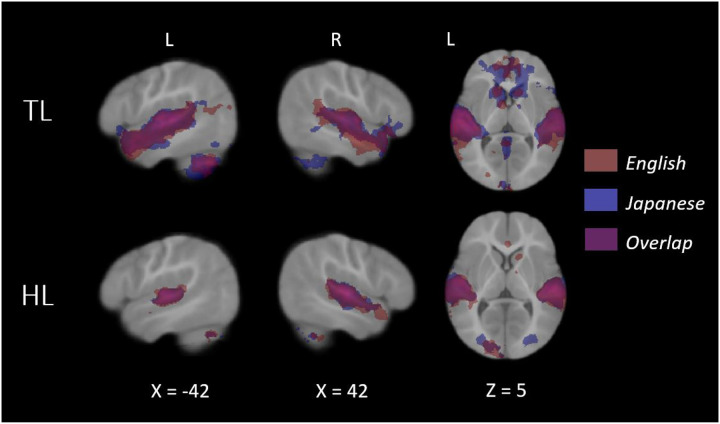
Legend not included with this version.

**Figure 2:**
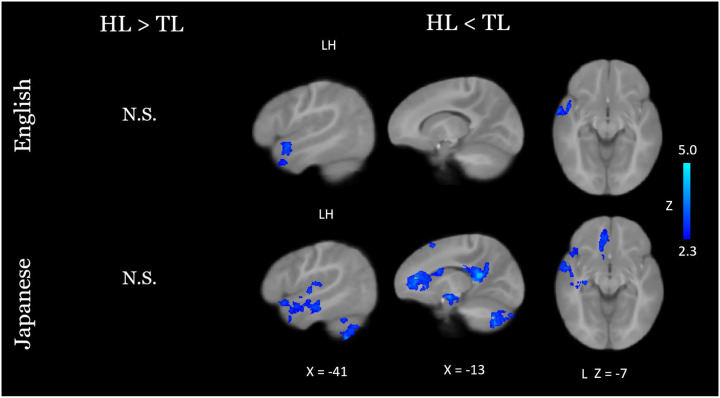
Legend not included with this version.

**Figure 3:**
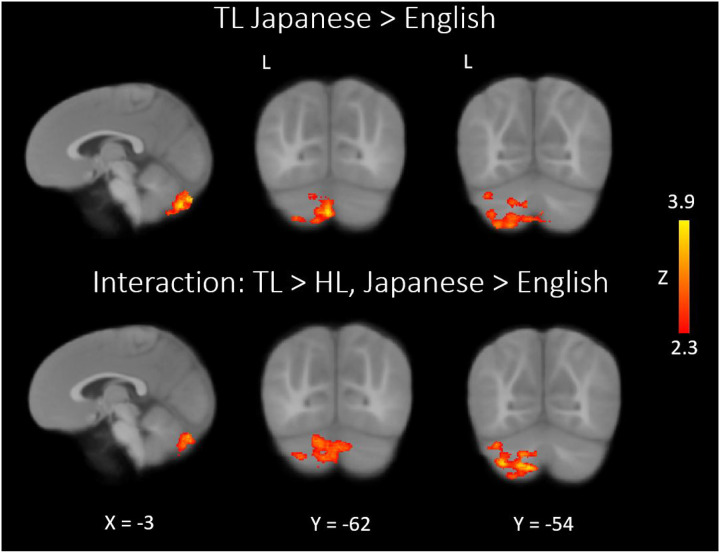
Legend not included with this version.

At nine months of age, both groups of infants recruited primary and secondary auditory processing regions in response to both the native and novel speech conditions (Fig. 1). Group comparisons showed that HL infants exhibited hypoactivity in response to each language condition, relative to TL infants, in left temporal areas associated with language (Fig. 2; see Table 2 for coordinate table). During the novel language condition only, compared to TL infants the HL group also showed hypoactivity in additional prefrontal (superior frontal and anterior cingulate gyri), parietal (left precuneus and posterior cingulate gyrus), and cerebellar (lobules VIIb and VIIIa, right VIIIb, and left crus I and II) regions, as well as the left hippocampus. Moreover, whereas the HL group did not show differential responses for the two language conditions, the TL group showed significantly greater cerebellar activation in the bilateral crus II, left lobule VIIb, left lobule VIIIa, and left lobule VI during the novel language condition relative to the native condition (Fig. 3). A very similar, largely overlapping cerebellar cluster was also present in the interaction contrast (TL >HL, Japanese > English; Fig. 3). Local activation peaks for the interaction were identified in the left Crus I and II, Lobules VIIb and VIIIb, and in the vermis (Table 2). All group differences held when controlling for potential confounds (sex, maternal education, and birth order) in post-hoc tests (see Table S1).

### ROI Analyses

#### Activation Strength.

**Figure 4:**
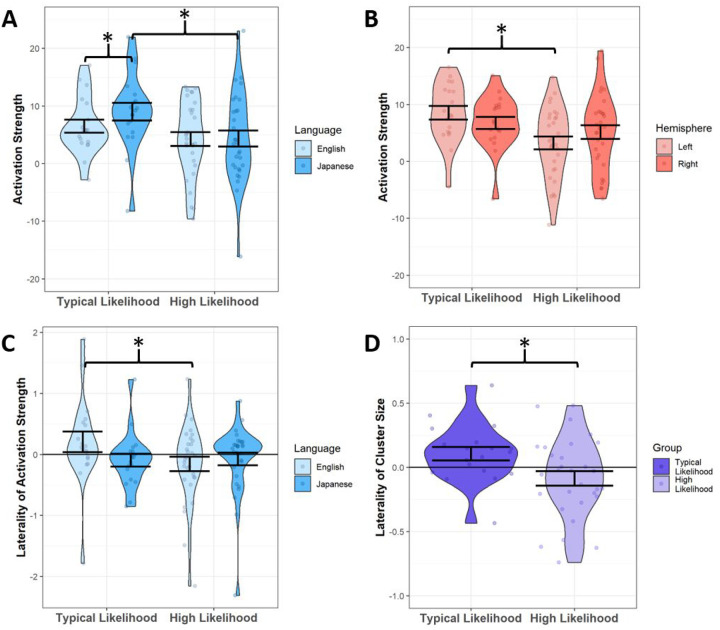
Legend not included with this version.

We found a significant interaction between likelihood group and language condition on activation collapsed across all ROIs and hemispheres (F(1, 26) = 4.82, p = 0.037; Fig. 4A). Post-hoc analyses showed a simple main effect of likelihood group in the novel language condition only (F(1, 33) = 6.589, p = 0.015), such that the TL group had significantly stronger activation for the novel language than the HL group did. We also saw a simple main effect of language condition for the TL group only (F(1, 9) = 5.292, p = 0.047), such that the TL group exhibited stronger activation during the novel language condition than to the native condition across ROIs. Together, these results indicate that the TL infants exhibit not only greater activation in response to language, but also greater neural differentiation between their native language and a foreign language. Additionally, there was a significant interaction between likelihood group and hemisphere (F(1, 26) = 4.619, p = 0.041; Fig. 4B) such that the TL group had significantly greater left-hemisphere activation across all ROIs and language conditions as compared to the HL group (F(1, 32) = 6.597, p = 0.015).

Next, when examining laterality indices of activation strength, we found a significant interaction between likelihood group and language condition (F(1, 36) = 7.921, p = 0.008; Fig. 4C). Post-hoc analyses showed that the TL group had stronger left-hemisphere activation during the native language condition than the HL group did, across ROIs (F(1, 40) = 6.427, p = 0.015).

#### Cluster Size.

When examining cluster size as a variable of interest, we found no significant differences between likelihood groups for either language condition. However, when examining the laterality index based on cluster size, we found a main effect of likelihood group (F(1, 34) = 7.754, p = 0.009) such that the TL group had significantly larger clusters of activation in the left hemisphere across language areas compared to the HL group (Fig. 4D).

## Discussion

In the present study, we examined neural responses to native and novel speech in order to investigate how the neural signatures of native language processing may differ in a sample of nine-month-old infants at high and typical familial likelihood for autism. As compared to the TL group, HL infants showed diminished neural responses in left temporal language areas for both their native language and a novel language. Furthermore, HL infants also showed no evidence of neural discrimination between the two languages, unlike the TL group, who showed a neural novelty response when presented with the unfamiliar language. In a subsequent *a priori* analysis that focused on canonical language regions, HL infants also showed patterns of atypically lateralized speech processing, in terms of both strength and spatial extent of neural activation. To our knowledge, this is the first study to use fMRI to directly index autism-associated atypicalities related to native language acquisition in infancy.

Consistent with prior literature showing reliable bilateral activation of temporal language areas in sleeping infants passively exposed to speech (25, 38, 56), here we observed activation in temporal language areas for both groups and for both the native and novel languages. In line with prior work examining speech processing in HL infants (36) and in toddlers with an ASD diagnosis (28, 30), we found that HL infants exhibited a general pattern of hypoactivation across both languages. Thus, we extend prior findings that have linked autism with diminished neural responses to speech in early development. Across both languages, HL infants reliably showed attenuated responses to speech in left temporal language areas. Prior work has shown that similar patterns of temporal hypoactivation predict poorer language skills in autistic toddlers (27, 28). During exposure to the novel language, HL infants also exhibited hypoactivity in additional brain areas related to attention, memory, and cognitive function, as well as in several cerebellar lobules. The limited responses in these regions suggest that novel social input may not elicit increased attention in HL infants as it does in the TL group. In other words, the greater engagement of these non-canonical language regions (e.g., anterior and posterior cingulate, medial prefrontal cortex, hippocampus) likely reflects increased attention that novel social stimuli implicitly elicit in the typically developing brain. Indeed, a “novelty preference” has been well established in the behavioral literature on late infancy (9), and is generally considered a marker of increased attention, social interest, and even active learning. In more recent behavioral language studies of 9- (57) and 10-month-old (58) infants, for example, the presence of a behavioral novelty preference was directly linked to larger vocabulary size.

Interestingly, compared to their TL counterparts, nine-month-old HL infants displayed no evidence of discrimination between the native and novel languages. The ability to differentiate between native and novel languages at the neural level can be viewed as a proxy for native language acquisition progress and, therefore, we interpret this lack of discrimination as a potential marker of diminished uptake of their native language. By contrast, TL controls showed differential patterns of neural activation, characterized by heightened brain responses to the novel language. Therefore, our observation of a novelty response at the neural level – in the TL group only – may be considered a marker of normative language acquisition as well as provide a potential indicator of early delayed or derailed language uptake in the HL group. Notably, this neural discrimination was most evident in the cerebellum, which has increasingly been acknowledged for its important role in language and social cognition (32, 54, 59) in addition to its crucial motor functions. In adults, not only is the cerebellum important for processing prosody and comprehending syntax (reviewed in LeBel & D’Mello (32)) – it is also a component of brain networks for reading in adults (60) and children (61), and lesions to the right lateral aspect are known to induce deficits in verbal fluency (60). Indeed, several regions where TL infants showed this neural novelty response – lobules VI, VIIb, and VIIIa – were previously identified as having a functional role in language according to a study that used a battery of tasks to map the functional topography of the cerebellum (54). Cerebellar crus I and II also responded more strongly during exposure to the novel language in the present study. This is especially interesting given that these areas of the posterior cerebellum are part of reciprocal circuits that project to prefrontal, temporal, and parietal language areas of the cortex (32). Crus I in particular is consistently implicated in language functions (see, e.g., (55)). Damage to the right crus I can cause disruptions in phonological fluency and reorganization of left frontal language areas (62). A recent, more relevant study also observed aberrant cortical functional connectivity with crus I in HL infants with delayed language (34).

The analysis conducted using the *a priori* region of interest (ROI) approach corroborated the results observed in the whole brain analysis. Specifically, the TL group responded significantly stronger to the novel language than to the native language, across all language ROIs tested, whereas the HL group showed similar activation strength in language ROIs across both languages. The significantly stronger activation to the novel language in the TL group provides further evidence that, at 9 months of age, a neural novelty response when processing unfamiliar linguistic input may be normative, and may indicate a level of attunement conducive to the uptake of novel social stimuli. Furthermore, using the ROI approach the HL group showed significantly weaker left-hemisphere activation across language ROIs during both languages compared to TL infants, directly in line with our earlier findings of weaker left temporal activity observed in the whole-brain analysis. While bilateral temporal language processing is present in typically-developing neonates soon after birth (25), the left hemisphere becomes increasingly specialized for native speech within the first few months of life (56, 63, 64) and this lateralization becomes stronger as language skills develop (65). Interestingly, one fNIRS study (66) found that native speech processing in newborns already shows some leftward bias in temporal areas, whereas the unfamiliar prosodic patterns of a novel language elicit a rightward bias. Indeed, our findings in this 9-month-old sample showed a lack of leftward lateralization in novel language processing for the TL group.

Importantly, our laterality analysis in language ROIs showed that, during the native English condition, specifically, HL infants had striking, atypical patterns of rightward lateralization with regard to neural activation strength. Additionally, the HL group exhibited larger clusters of activation in the right hemisphere than in the left during speech processing across both languages. Atypically right-lateralized speech processing is well documented in autistic individuals, from toddlers (29, 30) to adolescents (39) and even in adults (67). Therefore, this finding extends previously reported atypicalities to an earlier developmental age. A number of studies have linked greater rightward language lateralization with poorer language abilities in children and adults with ASD (68, 69). The existing literature suggests a general pattern in which an early rightward bias in language processing may be detrimental, but some studies in older individuals have found that volumetric (70) and functional (39) rightward lateralization in frontal areas predicted better language abilities in older ASD individuals. Thus, studies in older individuals suggest that some amount of rightward language lateralization may be compensatory in autism (29, 39). Further longitudinal investigations of language lateralization are needed to characterize the degree to which its autism-associated atypicalities are compensatory, as opposed to detrimental, throughout development.

Although prior studies have used EEG to uncover differences in native versus novel language processing in HL infants (71, 72), the present study is the first to investigate their neural substrates using fMRI. Prior fMRI studies of language processing in HL infants have compared speech against environmental sounds (36) or have investigated statistical word learning using an artificial language (38). Therefore, the native vs. novel paradigm utilized in the present study addresses an important gap in the literature: by comparing the neural correlates of native language processing against neural responses to another natural language, we are able to tap into the neural substrates subserving language learning, allowing us to more directly index the degree to which language learning has occurred.

### Limitations

We acknowledge several limitations of the present study. Foremost, the sample size, particularly in the TL group, was quite modest. Achieving a sample size suitable for analysis was challenging given that passive fMRI auditory paradigms frequently awaken sleeping participants and induce motion artifacts. Recruitment was especially challenging for the TL group: the discrepancy between groups is because the HL sample was largely recruited through UCLA’s Center for Autism Research & Treatment, which is tapped into a local network of families of children with ASD who are enthusiastic about autism research. The TL group was not recruited through any such network of families. We also experienced challenges in participant retention because of the COVID-19 pandemic, which caused sample attrition and the loss of behavioral and diagnostic follow-up data. Moreover, the groups were not matched on some variables that could introduce confounds: the TL group skewed significantly more female and had higher maternal education, and also had a substantial number of firstborn participants. However, our post-hoc analyses indicate that these findings held even when accounting for these potential confounding factors (Table S1). Additionally, the HL group is expected to be more developmentally heterogeneous than the TL group: some HL participants will later develop autism, while others will develop language delays, and still others will have no discernible atypicalities. Thus, our treatment of HL infants as a monolithic group is not optimal, but necessary due to the limited sample size and some participants missing diagnostic outcome. Another limitation, shared by virtually all infant neuroimaging studies, is that we were unable to collect data on sleep stage during the MRI scan. Thus, stimulus-evoked paradigms are generally unable to control for the potential confounding effect of sleep stage on neural activity. However, one study in ASD and TD toddlers (73) recently tested the effect of sleep stage (proxied by the amount of time between falling asleep and the scan start) on BOLD activity in primary auditory cortex. BOLD measures did not correlate with time between falling asleep and the start of the MRI, and similarly did not differ between discrete resting-state functional runs, when infants would presumably be in different sleep stages. Furthermore, a sizable body of work in infant participants shows that observing robust neural activation during sleep is not only possible, but well established (e.g., (25, 36, 38)). Future studies using native vs. novel language paradigms should also seek to collect longitudinal fMRI data at several stages of infant development. There is also a conspicuous gap in the native versus novel language processing literature in typical development, which is important for developing a baseline understanding of the neural correlates of native language acquisition. Doing this at the level of large-scale neuroimaging consortia would be especially impactful, as a large longitudinal study would produce a detailed understanding of normative developmental trajectories against which to compare atypical development.

## Conclusions

In sum, the present study extends prior work on the neurodevelopmental basis of language delay in autism by investigating how the neural signatures of native language processing differ in 9-month-old infants at high and typical familial likelihood for autism. HL infants showed diminished neural responses to speech in left temporal language areas during both languages, as well as an absence of neural differentiation between native and novel speech. We also extend prior reports of atypical rightward language lateralization in autism to this earlier developmental age. To our knowledge, this is the first study that uses fMRI to directly index autism-associated atypicalities related to native language acquisition in infancy. This work furthers our understanding of the neural correlates of language acquisition in autism, which is a critical first step to develop earlier, timelier interventions that could alleviate or mitigate language impairments seen in autistic children and HL siblings.

## Figures and Tables

**Table 1:** Participant demographics, behavioral assessments, and outcome assessments by likelihood group. Mean ± standard deviation is shown for continuous variables (age, motion metrics, and behavioral assessments).

	HL	TL	P (t or X^2^)
*N*	30	19	N/A
Sex (Female)	8 (26%)	13 (68%)	0.009**
Age (Months)	9.17 ± 0.31	9.08 ± 0.39	0.38
Race^1^			
White	15	12	0.83
Nonwhite	12	7	
Ethnicity^2^			
Hispanic	9	1	0.10
Non-Hispanic	21	17	
Maternal Education^3^			
No college	0	0	0.001***
Some college or bachelor’s degree	22	4	
Post-graduate education	7	14	
Birth Order^4^			
First	0	8	<0.001***
Not first	30	9	
Exposure to English (% time)	89.5 ± 16.6	92.4± 13.9	0.52
Mean Absolute Motion (mm)	0.21 ±0.21	0.36 ± 0.57	0.28
Mean Relative Motion (mm)	0.07 ± 0.04	0.11 ± 0.11	0.16
# Motion Outlier Volumes	1.07 ± 2.24	3.53 ± 8.94	0.25
MSELT-scores (12 months)			
Gross Motor	49.8 ± 9.5	48.8 ± 15.3	0.82
Fine Motor	59.5 ± 9.7	67.7 ± 7.8	0.004**
Visual Reception	56.7 ± 6.9	60.5 ± 7.7	0.10
Receptive Language	46.9 ± 8.5	53.2 ± 5.2	0.004**
Expressive Language	50.0 ± 12.4	51.8 ± 9.2	0.58
MSEL T-scores (36 months)			
Gross Motor	–	–	–
Fine Motor	51.5 ± 16.3	59.7 ± 12.9	0.08
Visual Reception	56.7 ± 18.0	66.5 ± 8.2	0.02*
Receptive Language	49.1 ± 14.2	63.0 ± 8.2	< 0.001***
Expressive Language	50.8 ± 12.9	61.3 ± 7.2	0.002**
Vineland (12 months)			
Communication	85.4 ± 21.5	100.3 ± 15.6	0.02*
Socialization	85.1 ± 24.3	86.8 ± 34.2	0.87
Vineland (36 months)			
Communication	95.2 ± 17.0	110.7 ± 8.8	< 0.001***
Socialization	92.9 ± 17.1	110.0 ± 8.5	< 0.001***
AOSI (12 months)	5.0 ± 2.9	3.6 ± 2.1	0.08
ADOS-2 (36 months)	2.7 ± 1.5	2.2 ± 2.2	0.45
Outcome Classification			
ASD	6	0	0.013*
TD	13	15	
Other Concerns	7	1	
MCDI (12 months)			
Words Comprehended	39.0 ± 49.8	66.5 ± 49.1	0.09
Words Produced	6.1 ± 9.9	4.1 ± 5.1	0.38
MCDI (36 months)			
Words Produced	460.3 ± 210.5	590.1 ± 94.6	0.02*
ESCS (12 months)			
IJA	14.0 ± 8.1	11.6 ± 7.0	0.37
RJA	4.4 ± 2.5	8.0 ± 2.9	< 0.001***

1 Race unknown for 3 HL participants.

2 Ethnicity missing for one TL participant.

3 Maternal education information missing for one HL and one TL participant.

4 Birth order information missing for 2 TL participants

Sample sizes for behavioral data: MSEL at 12 months: 18 TL, 26 HL; Vineland at 12 months: 12 TL, 23 HL; MCDI at 12 months: 17 TL, 25 HL; AOSI at 12 months: 15 TL, 27 HL; ESCS at 12 months: 14 TL, 22 HL; MSEL at 36 months: 15 TL, 26 HL; Vineland at 36 months: 16 TL, 24 HL; MCDI at 36 months: 15 TL, 21 HL; ADOS-2 at 36 months: 13 TL, 19 HL.

**Table 2:** Coordinate table for activation peaks within likelihood groups and language conditions, in comparisons between likelihood groups, and in comparisons between languages. Peaks Z > 3.1 reported. **TL:** typical likelihood; HL: high likelihood.

English > Silence									
		TL				HL			
Region	Hemisphere	Max Z	X	Y	Z	Max Z	X	Y	Z
Superior temporal gyrus	L	6.08	−51	−14	3	6.25	−47	−10	4
	R	5.73	47	−10	2	7.3	49	−15	2
Middle temporal gyrus	L	5.85	−53	−9	−2	4.96	−58	−13	−1
	R	4.99	56	−6	−9	5.73	53	−21	−3
Middle temporal pole	L	4.53	−39	14	−19				
	R	4.13	43	5	−16	3.33	44	10	−16
Superior temporal pole	L	4.46	−41	11	−16				
	R	3.8	41	15	−11	4.18	53	3	−8
Orbitofrontal cortex (medial)	L	3.48	−4	49	−7				
	R	3.56	4	40	−8				
Rectus gyrus	L	3.41	1	44	−12				
	R	3.48	7	40	−12				
Orbitofrontal cortex (superior)	R	3.22	10	46	−9				
Precuneus	L	3.45	−5	−41	27				
	R	3.63	0	−44	28				
Middle cingulate gyrus	R	3.25	1	−40	25				
Superior frontal gyrus (medial)	L	3.22	−9	44	6				
	R	3.42	9	44	8				
Posterior cingulate gyrus	L	3.23	−2	−37	7				
	R	3.39	3	−42	17				
Caudate	L	3.3	−10	15	9				
	R	3.25	9	13	−5	3.39	12	17	−4
Olfactory cortex	R	3.15	4	14	0	3.66	18	13	−8
Parahippocampus	L	3.3	16	12	−7				
Cuneus	L					3.53	−2	−77	24
	R	3.32	0	−86	12				
Amygdala	L	3.13	−14	−3	−11				
Inferior temporal gyrus	L	3.26	−35	4	−23				
Fusiform gyrus	L	3.28	−26	−67	−19				
Superior frontal gyrus (dorsal)	L	3.22	−13	47	18				
Lingual gyrus	L	3.26	−8	−56	−8	4.41	−11	−65	−20
	R					3.16	4	−74	−23
Putamen	L	3.23	−13	7	9				
	R	3.12	13	12	−5				
Orbitofrontal cortex (inferior)	R	3.18	39	19	−6				
Angular gyrus	L	3.11	−49	−49	27				
Superior occipital gyrus	L	3.11	−16	−76	−2	3.28	−16	−80	3
Calcarine cortex	L					3.67	−11	−81	−1
Middle occipital gyrus	L					3.7	−28	−69	−5
Crus I	L	5.73	−42	−51	−30	4.72	−20	−63	−28
	R	5.48	27	−60	−34	4.24	35	−45	−34
Lobule VI	R	5.07	19	−59	−23				
Crus II	L	4.05	−27	−60	−37	3.15	−15	−66	−33
	R	4.48	4	−69	−34	4.72	3	−71	−27
Lobule Villa	L	3.2	−23	−44	−40				
	R	4.08	22	−37	−44				
Lobule Vllb	L	3.75	−13	−57	−42				
	R	4.04	29	−43	−42	3.21	30	−42	−43
Lobule VI	L	4.01	−29	−47	−27	4.99	−13	−65	−21
	R					4.02	21	−64	−23
Lobule V	L	3.59	−22	−20	−29				
	R	3.99	13	−34	−18				
Vermis	--	3.87	3	−62	−18	5.53	2	−64	−26
Japanese > Silence									
		TL				HL			
Region	Hemisphere	Max Z	X	Y	Z	Max Z	X	Y	Z
Superior temporal gyrus	L	6.21	−48	−7	3	6.47	−40	−19	3
	R	5.8	49	−9	2	7.35	44	−15	1
Middle temporal gyrus	L	5.98	−48	−11	0	4.74	−53	−9	−2
	R	4.73	55	−6	−9	5.89	55	−17	−5
Superior temporal pole	L	4.4	−37	22	−17				
	R	4.49	49	4	−4	4.44	55	2	1
Middle temporal pole	L	3.86	−26	21	−26				
	R	3.47	37	9	−20	3.18	47	6	−17
Precuneus	L	3.67	−2	−44	4				
	R	3.77	4	−38	6	3.14	6	−68	42
Calcarine cortex	L	3.49	−2	−54	2	3.16	−6	−81	−1
	R	3.67	0	−48	2				
Posterior cingulate gyrus	L	3.56	0	−34	15				
	R	3.63	3	−35	13				
English > Silence									
		TL				HL			
Region	Hemisphere	Max Z	X	Y	Z	Max Z	X	Y	Z
Middle cingulate gyrus	L	3.46	−6	−24	23				
	R	3.33	2	−43	19				
Caudate	L	4.08	−6	13	−1				
	R	3.93	7	13	4				
Olfactory cortex	L	3.49	−19	9	−9				
	R	3.11	5	10	−17				
Rectus gyrus	L	3.25	−12	16	−7				
	R	3.5	6	41	−12				
Putamen	L	3.24	−13	11	−3				
Pallidum	L	3.17	−8	3	2				
	R	3.19	11	0	1				
Orbitofrontal cortex (medial)	L	3.91	−3	38	−4				
	R	3.55	1	45	1				
Anterior cingulate gyrus	L	3.51	0	40	1				
	R	3.56	12	38	5				
Superior frontal gyrus (medial)	L	3.62	−1	30	45				
	R	3.31	4	49	2				
Supplementary motor area	L	3.37	0	5	50				
	R	3.4	1	13	49				
Lingual gyrus	L	3.84	−4	−63	−13	3.26	−11	−63	−18
	R	3.74	2	−64	−18	3.41	1	−67	−18
Parahippocampus	L	3.4	−13	−3	−12				
	R	3.67	13	−17	−19				
Amygdala	L	3.31	−12	1	−11				
Hippocampus	L	3.47	−19	−10	−18				
Fusiform gyrus	L	3.44	−28	−61	−20	3.68	−20	−43	−15
	R	3.88	13	−65	−20	3.2	24	−41	−19
Inferior occipital gyrus	L	3.64	−28	−66	−14				
	R	3.11	17	−82	−10				
Operculum	L	3.58	−36	−57	−22				
	R	3.21	57	−7	8				
Thalamus	L	3.57	−5	−6	13				
	R	3.14	0	−13	10				
Orbitofrontal cortex (superior)	L	3.14	−7	43	−21				
	R	3.43	11	45	−16				
Orbitofrontal cortex (inferior)	L								
	R	3.4	33	22	−5				
Cuneus	L	3.27	−6	−81	5	3.48	−6	−80	14
	R	3.38	−1	−79	25	3.3	13	−71	33
Middle frontal gyrus	L	3.34	−22	34	16				
	R	3.2	22	42	7				
Superior frontal gyrus (dorsal)	L	3.18	−16	43	8				
	R	3.33	16	42	33				
Inferior frontal gyrus (triangular)	R	3.26	31	23	3				
Postcentral gyrus	L	3.24	−58	−7	12				
Heschl’s gyrus	L	3.21	−29	−24	11				
Inferior temporal gyrus	L	3.21	−28	−8	−32				
	R					3.18	23	−64	−22
Inferior frontal gyrus (opercular)	R	3.11	27	13	27				
Superior occipital gyrus	L					3.41	−17	−75	16
	R					3.77	14	−76	32
Middle occipital gyrus	L					3.1	27	−63	−23
	R					3.62	23	−69	14
Superior parietal gyrus	R					3.32	17	−60	45
Crus I	L	7.32	−24	−57	−29	3.55	−26	−58	−26
	R	5.77	9	−67	−27	4.86	26	−65	−31
Crus II	L	5.96	−4	−65	−37	3.53	−6	−73	−32
	R	6.36	−11	−60	−40	3.81	15	−69	−34
Lobule VI	L	6.25	−21	−53	−29	4.01	−11	−65	−20
	R	6.1	10	−64	−22	4.25	23	−50	−21
Lobule Villa	L	5.37	−29	−36	−47				
	R	6.06	22	−41	−45				
Lobule Vllb	L	5.76	−14	−56	−42				
	R	5.92	29	−43	−42				
Vermis	--	5.22	−1	−64	−19	3.19	−1	−64	−18
Lobules I - IV	L	3.37	−11	−23	−21				
	R	4.09	13	−17	−20				
Lobule V	L	4.07	−21	−20	−28	3.63	−18	−31	−19
	R	3.48	23	−25	−26	3.25	5	−54	−13
Lobule VIIIb	R	3.68	16	−40	−42				
Lobule IX	R	3.37	7	−32	−44				
Lobule X	L	3.32	−21	−23	−34				

TL>HL, English					
Region	Hemisphere	Max Z	X	Y	Z
Inferior temporal gyrus	L	3.74	−34	9	−26
Superior temporal gyrus	L	3.67	−41	7	−9
Temporal pole	L	3.57	−35	10	−24
Middle temporal gyrus	L	3.47	−36	4	−18
TL > HL, Japanese					
Region	Hemisphere	Max Z	X	Y	Z
Posterior cingulate gyrus	L	4.98	−11	−37	13
Precuneus	L	3.98	−3	−37	2
Hippocampus	L	4.09	−12	−3	−11
Middle temporal gyrus	L	4.05	−37	7	−19
Superior temporal gyrus	L	4.04	−43	−7	3
Anterior cingulate gyrus	L	3.73	−12	34	12
	R	3.3	14	35	12
Superior frontal gyrus (medial)	L	3.42	−4	26	35
	R	3.63	12	40	6
Superior frontal gyrus (dorsal)	L	3.25	−16	25	41
Lobule Vllb	L	4.26	−19	−52	−41
	R	3.15	29	−47	−44
Crus II	L	3.77	−41	−43	−43
Lobule Vllla	L	3.7	−25	−39	−43
	R	3.25	23	−38	−43
Crus I	L	3.56	−38	−39	−26
Lobule VIIIb	R	3.1	21	−35	−47
TL Japanese > English					
Region	Hemisphere	Max Z	X	Y	Z
Crus II	L	3.88	−5	−71	−32
	R	3.31	3	−75	−32
Lobule VIIb	L	3.35	−32	−37	−45
Lobule VIIIa	L	3.3	−16	−53	−47
Lobule VI	L	3.12	−18	−50	−27
TL > HL, Japanese > English (Interaction)					
Region	Hemisphere	Max Z	X	Y	Z
Lobule VIIb	L	3.87	−17	−52	−40
Crus II	L	3.78	−29	−52	−38
Lobule VIIIb	L	3.75	−12	−53	−41
Vermis	--	3.62	3	−63	−29
Crus I	L	3.59	−30	−52	−35

## Data Availability

The dataset(s) supporting the conclusions of this article are available via the National Institutes of Mental Health Data Archive. Researchers can submit a Data Access Request to access de-identified human subjects data at https://nda.nih.gov/.

## Abbreviations

ASD: Autism Spectrum Disorder
HL: High Likelihood for Autism
TL: Typical Likelihood for Autism
fMRI: Functional Magnetic Resonance Imaging
MSEL: Mullen Scales of Early Learning
ELC: Early Learning Composite
VABS-II: Vineland Adaptive Behavior Scales, Second Edition
MCDI: MacArthur-Bates Communicative Development Inventory
ESCS: Early Social Communication Scales
RJA: Responding to Joint Attention
AOSI: Autism Observation Scale for Infants
ADOS-2: Autism Diagnostic Observation Schedule - 2nd Edition
ROI: Region of Interest
